# The Role of MicroRNAs in the Chemopreventive Activity of Sulforaphane from Cruciferous Vegetables

**DOI:** 10.3390/nu9080902

**Published:** 2017-08-19

**Authors:** Christopher Dacosta, Yongping Bao

**Affiliations:** Norwich Medical School, University of East Anglia, Norwich NR4 7UQ, UK; Christopher.A.Dacosta@gmail.com

**Keywords:** broccoli, cancer, cruciferous vegetable, microRNA, sulforaphane, isothiocyanate

## Abstract

Colorectal cancer is an increasingly significant cause of mortality whose risk is linked to diet and inversely correlated with cruciferous vegetable consumption. This is likely to be partly attributable to the isothiocyanates derived from eating these vegetables, such as sulforaphane, which is extensively characterised for cytoprotective and tumour-suppressing activities. However, its bioactivities are likely to extend in complexity beyond those currently known; further insight into these bioactivities could aid the development of sulforaphane-based chemopreventive or chemotherapeutic strategies. Evidence suggests that sulforaphane modulates the expression of microRNAs, many of which are known to regulate genes involved at various stages of colorectal carcinogenesis. Based upon existing knowledge, there exist many plausible mechanisms by which sulforaphane may regulate microRNAs. Thus, there is a strong case for the further investigation of the roles of microRNAs in the anti-cancer effects of sulforaphane. There are several different types of approach to the wide-scale profiling of microRNA differential expression. Array-based methods may involve the use of RT-qPCR or complementary hybridisation probe chips, and tend to be relatively fast and economical. Cloning and deep sequencing approaches are more expensive and labour-intensive, but are worth considering where viable, for their greater sensitivity and ability to detect novel microRNAs.

## 1. Colorectal Cancer

Colorectal cancer is a major and increasingly common cause of morbidity and premature death. Its global incidence was 1.4 million in 2012, according to the World Health Organization, and is rising, making it the third most commonly diagnosed cancer [1]. In 2015, 774,000 mortalities were directly attributable to colorectal cancer—a figure 58% higher than in 1990 [2]. It is rare for tangible symptoms of colorectal cancer to present until it has already progressed to an advanced (usually terminal) stage, at which currently available treatments are unable to provide a cure [3]. Therefore, strategies for limiting the disease burden from colorectal cancers must include improvements to early-stage diagnosis in asymptomatic individuals, as well as new preventive initiatives.

Reported incidence correlates positively with economic development and/or the adoption of “Western” dietary patterns [4]. Increasing rates of chronic diseases such as cancers are inevitable in countries experiencing rising living standards, partly due to reduced rates of premature death from communicable diseases. Economic development also tends to facilitate improvements to public and/or affordable private healthcare, leading to improved diagnosis. Nevertheless, there is compelling evidence to suggest that commonly associated dietary and lifestyle changes have a significant impact upon the development of colorectal cancer [5]. Particularly, one’s risk of developing colorectal cancer is believed to be increased by obesity and a high intake of red meat and/or alcohol and reduced by a fibre-rich diet abundant in fruits and vegetables and a physically active lifestyle, according to guidelines published by Bowel Cancer UK [6]. The apparently strong connections between diet and colorectal cancer risk are unsurprising, given the inevitable exposure of the colorectal tissues to ingested compounds and products of the gut microbiota. The apparently protective effects of diets rich in plant-based foods are believed to be largely attributable to the phytochemicals found in them, many families of which have been studied regarding their direct biological (often cytoprotective) activity—both in vitro and in vivo—since the middle of the twentieth century. 

## 2. Cruciferous Vegetables

Epidemiological studies have revealed a particular inverse correlation between the intake of cruciferous vegetables and colorectal cancer risk; one stronger than that between the latter and the intake of other vegetables [7,8]. Cruciferous vegetables refer to those of the Brassicaceae family and include broccoli, cabbage, and Brussel sprouts. Particular to this plant family are glucosinolates—a group of compounds endogenously synthesised and derived from glucose and amino acid residues. Upon the rupture of plant cells—such as occurs from the consumption of the vegetables or from parasitic attack—the glucosinolates are able to be hydrolysed by endogenous myrosinase enzymes. Intact plant tissue separates glucosinolates from myrosinase enzymes by compartmentalising the former in S-cells [9] and the latter in myrosin cells [10]. Only upon cell rupture are the myrosinase enzymes able to hydrolyse the glucosinolates. Several types of compound are potentially formed, including isothiocyanates, thiocyanates, and nitriles [11]. 

## 3. Sulforaphane

**Figure 1:**
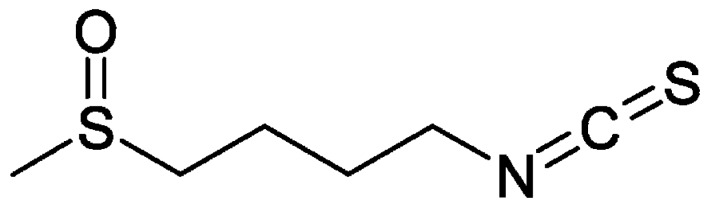
The molecular structure of 1-isothiocyanato-4-(methylsulfinyl)butane), also known as sulforaphane.

Isothiocyanates are to date the most-studied and best-characterised of known glucosinolate-hydrolysis-derived products in terms of their bioactivity. They are believed to play a defensive role in the plants via their cytotoxic effects on microorganisms and small parasitic animals, but to be directly beneficial to human health via broad anti-inflammatory and antioxidant effects, and thus are able to help inhibit the development of cancers [12], cardiovascular diseases [13], and osteoarthritis [14,15]. Broccoli is particularly high in a particular glucosinolate called glucoraphanin, whose myrosinase-mediated hydrolysis generates an isothiocyanate called sulforaphane (SFN, 1-isothiocyanato-4-(methylsulfinyl)butane), the structure of which is depicted in Figure 1.

**Figure 2:**
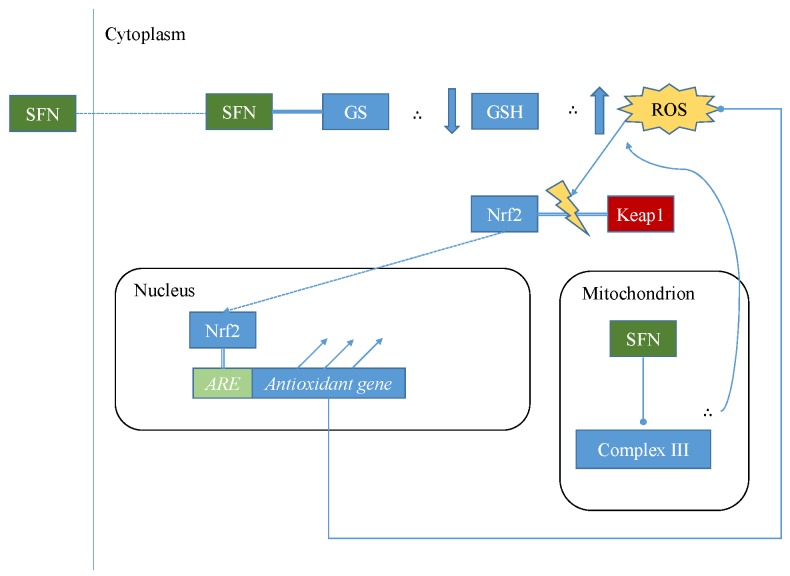
An illustration of the mechanisms by which sulforaphane (SFN) can induce Nrf2. SFN depletes intracellular GSH by forming complexes with it, and also inhibits complex III of the mitochondrial respiratory chain, thus increasing ROS levels. This acute increase in ROS causes Nrf2 to dissociate from Keap1, thereby enabling it to enter the nucleus where it transcriptionally activates antioxidant genes via *ARE* binding.

A multitude of experiments in vitro and in vivo reportedly demonstrate the ability of SFN to both defend healthy cells against chemical and/or radiation-induced carcinogenesis [16,17,18] and to inhibit the proliferation, migration, invasive potential and survival of tumour cells [19,20,21]. It is likely that the former, cytoprotective function of SFN is largely attributable to the induction of nuclear factor (erythroid-derived 2)-like 2 (Nrf2), resulting from the separation of cytoplasmic Nrf2 from Kelch-like ECH-associated protein 1 (Keap1), as illustrated in Figure 2. This allows Nrf2 to enter the nucleus, where it transcriptionally activates various genes, including those coding for antioxidant proteins such as thioredoxin reductase 1 [22] and uridine diphosphate (UDP)-glucuronosyltransferases [23]. These antioxidant proteins act to reduce reactive oxygen species (ROS) levels. ROS can react non-specifically with and thereby damage macromolecules such as lipids, proteins, nucleic acids and carbohydrates, promoting DNA damage–associated mutation and inflammatory signaling linked to age-related decline of function and the pathogeneses of chronic conditions such as atherosclerosis, neurodegenerative diseases and cancers. Therefore, a hypothesis popular in the middle of the twentieth century demonised ROS as toxic metabolic by-products, whose elimination could even halt the “ageing process” [24]. However, it has since been proven that ROS are vital for life and health, as essential components of cell-signaling pathways. For example, the glucose-induced secretion of insulin by β-pancreatic cells is dependent upon glucose-induced ROS generation [25]. Additionally, hydrogen peroxide binds to the regulatory domains of protein kinase C in a manner that promotes cell proliferation and inhibits apoptosis [26].

**Figure 3:**
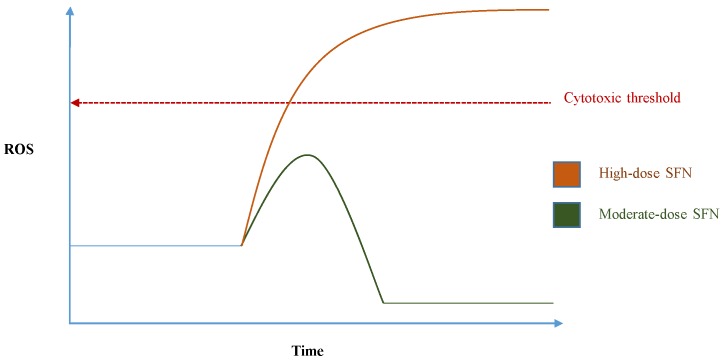
A sketch illustrating the hormetic effects of sulforaphane on cells with regards to oxidative stress, in that net reduction of oxidative stress is observed upon exposure to moderate doses, whereas increased oxidative stress and/or cytotoxicity occurs from high exposure.

SFN is commonly referred to as an “antioxidant” based on its widely demonstrated ability to help protect cells against oxidative stress at low-to-moderate doses [27]. However, SFN actually has an acute pro-oxidant effect in cells, largely by depleting intracellular glutathione due to the formation and export of SFN-glutathione complexes [28]. SFN can also increase mitochondrial ROS generation by inhibiting complex III of the mitochondrial respiratory chain, which causes the accumulation of ubisemiquionine, from which molecular oxygen receives electrons, resulting in the formation of superoxide and hydrogen peroxide [29]. SFN-induced acute oxidative stress is widely believed to be a significant driver of SFN-mediated Nrf2 induction, in addition to the SFN-mediated inhibition of p38 mitogen-activated protein kinase (MAPK), whose phosphorylation of Nrf2 inhibits Nrf2-Keap1 dissociation [30]. At low-to-moderate doses, the ensuing antioxidant response tends to outweigh those of the initial oxidative stress in redox terms, leading to a net protection against oxidative stress [31]. This is believed to be largely responsible for SFN’s cytoprotective potential in healthy cells. However, very high doses of SFN can be cytotoxic if the pro-oxidant effects induce significant macromolecular damage and/or ROS-mediated apoptosis before the mounting and execution of a sufficient antioxidant response, as illustrated by the sketch in Figure 3. This probably underlies the toxicity of SFN towards microorganisms and parasitic insects, as well as its observed abilities to inhibit tumour cell survival and metastasis [32]. The redox-modulating effects of SFN can thus be described as an example of hormesis. Hormesis is an ancient concept long characterised in various literature for medicinal and/or poisonous herbs—and more recently in scientific literature for nutrients, phytochemicals, and pharmaceutical drugs. In hormesis, low dose-exposure to a particular substance or stimulus has a net beneficial impact (in the case of sulforaphane, oxidative stress-inhibiting) upon the host that are consequential to the protective response it initiates. On the other hand, the net effects of higher dose-exposure are opposite and adverse (in the case of sulforaphane, oxidative stress-inducing) [33]. Doses obtainable from the consumption of cruciferous vegetables by humans are far below the cytotoxic threshold, thus tend to confer either neutral or cytoprotective effects. For example, one study has demonstrated plasma SFN concentrations to reach 10µM following the consumption of broccoli sprouts by study participants [34], which is a concentration demonstrated as non-cytotoxic, Nrf2-inducing and cytoprotective in various cell lines and systems.

Clearly, the induction of Nrf2 in healthy cells tends to be desirable from an anti-cancer perspective due to the inhibition of potentially carcinogenic ROS-induced mutation. In tumour cells, however, it can enhance their survival and proliferation, and make them more resistant to cytotoxicity-dependent anti-cancer therapies [35]. Nrf2 hyperactivation is in fact a marked feature of some cancers and a significant contributor to their aggressiveness. Thus, it has even been speculated that cytoprotective doses of SFN might be detrimental to patients undergoing treatment for advanced cancers, due to the induction of Nrf2 in tumour cells [36]. However, it is important to note that Nrf2 induction does not necessarily boost cell proliferation—particularly in early-stage tumour cells—and has in fact been reported to repress the proliferation of lung-cancer cells by inducing the breakdown of polyamines [37], whilst even to be responsible for the anti-proliferative effects of allicin in HCT-116 cells [38]. Nrf2 can also upregulate the surface expression of IL-17D, which in a systemic context could facilitate natural killer cell-mediated cell death in tumours [39]. Therefore, it is apparent that the interactions between SFN and redox status, and between redox status and carcinogenesis, are complicated.

## 4. MicroRNAs

ROS are not the only potential means by which high-dose SFN could repress the survival, proliferation, and metastatic characteristics of tumour cells. SFN can induce tumour-suppressing epigenetic changes by directly inhibiting histone deacetylases [40], some of which have a tendency to be aberrantly upregulated in colorectal cancer, thereby repressing various tumour suppressor genes at the transcriptional level via chromatin deacetylation [41]. However, another potential and less comprehensively studied means by which SFN may interact with cancers is the modulation of microRNA (miRNA) expression.

MiRNAs are small non-coding RNAs that are typically 18-25 nucleotides in length and that originate from various genetic loci such as the exons or introns of protein-coding genes and long non-coding exonic clusters (arrays). They post-transcriptionally regulate the expression of at least 30% of protein-coding genes in humans [42] by modulating messenger RNA (mRNA) translation, thus playing major roles in human development and health. Unsurprisingly, major roles for miRNAs in carcinogenesis are frequently reported [43], which is interesting in light of the apparent potential for SFN to modulate miRNA expression in several colorectal cell lines [44]. This leads to reasonable assumption that miRNA modulation has a role to play in SFN’s complex interactions with colorectal cancer.

### 4.1. Biogenesis

**Figure 4:**
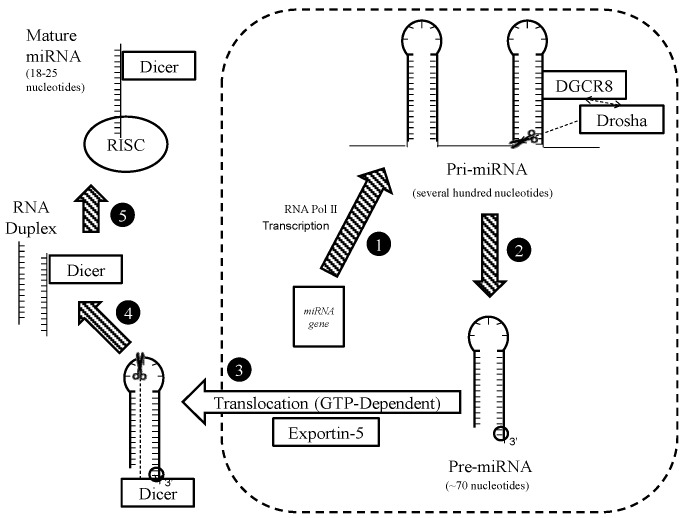
A diagram illustrating the canonical pathway of miRNA expression in animal cells. (1) A genetic locus is transcribed by RNA polymerase II, producing a pri-miRNA, which is several hundred nucleotides long. (2) The pri-miRNA is bound by DGCR8, which recruits Drosha, which cleaves the pri-miRNA into pre-miRNAs that are about 70 nucleotides long and have 2-nucleotide overhangs at their 3’ ends. (3) The 3’ 2-nucleotide overhangs are recognised by Exportin 5, which uses GTP to transport them from the nucleus to the cytoplasm. (4) Dicer recognises the same 3’ overhang and makes a nick in the loop region of the pre-miRNA, generating an imperfectly paired linear RNA duplex, each strand of which bears a 3’ 2-nucleotide overhang. (5) The linear RNA duplex is unwound; one strand remains associated with Dicer as the mature miRNA, and becomes part of a RISC upon association with AGOs.

The canonical pathway of miRNA biogenesis begins with the RNA polymerase II-mediated transcription of a long 5’-capped and 3’-polyadenylated transcript (the pri-miRNA), which is subsequently cleaved by DiGeorge Syndrome Critical Region 8 (DGCR8) to form products with distinctive hairpin-loop structures and 3’ 2-nucleotide overhangs (the pre-miRNAs) (Figure 4). The overhangs are recognised by exportin 5, which subsequently exports the pre-miRNAs to the cytoplasm where the same overhangs are recognised by Dicer [45]. A pre-miRNA may alternatively be formed by the conversion—by the lariat debranching enzyme (Ldbr)—of an intronic tract released upon the maturation of a protein-coding mRNA [46]. Dicer cleaves pre-miRNAs in their loop regions to generate linear duplexes, which are then unwound, and one strand from each of which remains Dicer-bound, then together with Dicer and Argonaute (AGO) proteins becomes part of an RNA-induced silencing complex (RISC) [45]. These single-stranded Dicer-bound RNAs are the mature miRNAs. Mature miRNAs can alternatively be generated non-canonically via the direct processing of a pre-miRNA by AGO2 followed by covalent modification and/or trimming [47]. 

### 4.2. Notes on Nomenclature

From each pre-miRNA, two mature miRNAs may be formed; one from each arm of the hairpin-loop motif. It was once assumed that only one of these tended to be functional (typically that with lower stability at the 5’ end), and that that from the other arm was degraded. Therefore—of the two mature miRNAs potentially formed from the processing of pre-miR-29b—the “dominant” product would have been called “miR-29b” whilst the other would have been termed “miR-29b*”. However, as evidence against this phenomenon being true in the majority of cases accumulated, miRBase nomenclature was changed such that the “*” suffix was dropped, and mature miRNAs were appended with “-5p” or “-3p” suffixes to denote the pre-miRNA arm of origin. According to this nomenclature, any mature miRNA names lacking a suffix are assumed to denote the only pre-miRNA processing product thus far identified. This updated miRBase nomenclature is used throughout this review.

### 4.3. Activities

The canonical mechanism of miRNA-mediated repression begins with the interaction of a 6–8 nucleotide seed region at the 5’ end of the miRNA with a locus or loci in the 3’-untranslated region (3’-UTR) of the mRNA, with which it is at least partially complementary in sequence. The RISC represses the translation of and/or degrades the bound mRNA, although the triggering of degradation is believed to be relatively rare in mammals, and to require perfect complementarity between the miRNA seed region and mRNA 3’-UTR [48]. AGO2 is the only AGO in mammals thus far shown to possess endonuclease activity [49]. Since only partial complementarity is required for translational repression, any given miRNA has the potential to target many different mRNA transcripts, whilst any given mRNA is potentially targeted by a multitude of miRNAs.

**Figure 5:**
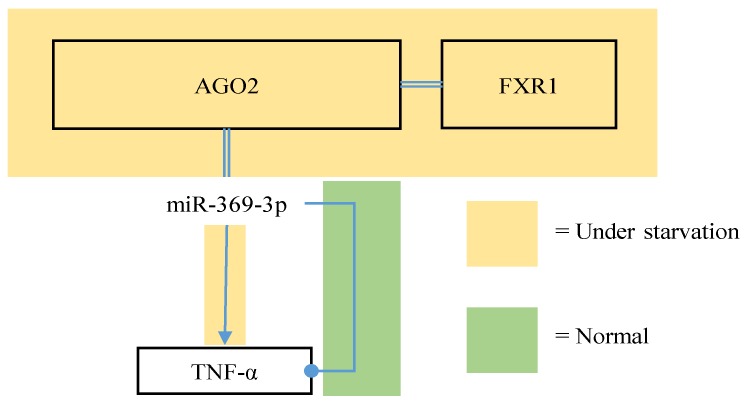
A diagram illustrating the ability of miR-369-3p to upregulate its target TNF-α under starvation, but to conversely repress it under normal conditions.

Although typically characterised as translational repressors, there are contexts in which an miRNA may conversely upregulate the translation of its target. For example, miR-369-3p promotes the translation of its target—tumour necrosis factor-α (TNF-α)—under serum-starved conditions, but represses it under normal conditions [50] as illustrated in Figure 5. This is facilitated by the association of the miRNA with AGO2 specifically, which itself is facilitated by the association of fragile X mental retardation-related protein 1 (FXR1) with AGO2 [50]. 

Hypothetically, serum-starvation could impact the solubility and/or localisation of AGO2-FXR1 complexes in a manner that promotes their association with miR-369-3p. The TNF-α transcript has an AU-rich element toward the 3’ end, which is required for its miRNA-mediated translational upregulation [50]. MiR-10a-5p was demonstrated to bind immediately downstream of 5’-oligopyrimidine motifs present in the 5’-UTRs of ribosomal protein mRNAs, and consequently upregulate their translation under amino acid starvation, in E14 ES mouse embryonic stem cells [51]. It has been proposed that miRNAs have a tendency to repress the translation of their targets in dividing cells, but conversely promote such in quiescent cells [52]. The apparent dependence of miRNA-mediated effects on nutritional status could be important when considering the roles of miRNAs at different stages of carcinogenesis, and in the context of certain therapies. Some miRNAs have been demonstrated to have RISC-independent effects, such as acting as decoys for RNA-binding proteins. For example, miR-328-3p acts as a decoy for the hnRNP E2 RNA-binding protein—which typically represses the transcription of a tumour-suppressing myeloid differentiation factor, CCAAT/enhancer-binding protein α—in chronic myeloid leukaemia [53]. MiR-328-3p thereby de-represses this tumour suppressor gene, and is demonstrably under-expressed in chronic myeloid leukaemia [54].

### 4.4. IsomiRs

Both at the cleavage of pri-miRNAs to form pre-miRNAs, and of the latter to form mature miRNAs, the Drosha and Dicer enzymes do not necessarily cleave at a precise locus, but have the potential to “slip” by several nucleotides in either direction [55]. Therefore, the mature miRNA sequences catalogued in miRBase do not necessarily represent specific RNAs of fixed sequence, but rather consensus sequences of distributions of isoforms that vary from the consensus in the form of having additional or missing nucleotides at either end. Such variant isoforms are called isomiRs. Perhaps the isomiR phenomenon evolved in cases where several isomiRs of a given miRNA all target a given mRNA in a desirable fashion, but affect different undesirable “off-target” mRNAs—i.e., the presence of multiple isomiRs could provide a mechanism for intensifying the desirable regulation of a specific mRNA target, whilst diffusing undesirable side-effects on other mRNAs [56].

## 5. Linking Sulforaphane, MicroRNAs, and Colorectal Cancer

A Google Scholar search was performed on 17th February 2017, using the following search string: intitle:microrna|mirna|micrornas|mirnas|”mir-“intitle:colorectal|colon|rectal|bowel intitle:cancer|tumour|tumor|cancers|tumours|tumors|carcinogenesis|tumorigenesis. This revealed existing reports of 144 miRNAs with apparent functions in colorectal cancer—of these, 85 were apparently tumour suppressive, 45 oncogenic, and 14 ambiguous in that reports of both oncogenic and tumour-suppressive function were found. Some examples of each are listed in Table 1, along with reported target genes.

The apparent involvement of many miRNAs in colorectal cancer is unsurprising, given the major roles of miRNAs in human health and development. There is also existing evidence to suggest that miRNA expression can be modulated in non-cancerous colonic cell lines by SFN. For example, Slaby et al. reported that SFN appeared to upregulate miR-9-3p, miR-23b-3p, miR-27b-5p, miR-27b-3p, miR-30a-3p, miR-135b-3p, miR-145-5p, miR-146a-5p, miR-342-3p, miR-486-5p, miR-505-3p, miR-629-5p, and miR-758-3p, but to downregulate miR-106a-3p, miR-155-5p, and miR-633-3p [44]. Two cell lines—normal derived colon mucosa 460 (NCM460) and normal derived colon mucosa 356 (NCM356)—were used, and TaqMan Low Density qPCR Arrays were used to profile the differential expression of 754 human miRNAs following 48 h SFN treatment [44]. This is a convincing indicator that SFN is able to modulate miRNA expression in colorectal cells, although it is important to note that the miRNAs reported as differentially expressed were not confirmed as so by additional assays. Also, only non-cancerous cell lines and a single time point post-treatment were studied, whereas the effects in cancerous colonic cells might differ substantially from those in their non-cancerous counterparts, and certain miRNAs may be transiently modulated at earlier time points in response to SFN treatment. 

**Figure 6:**
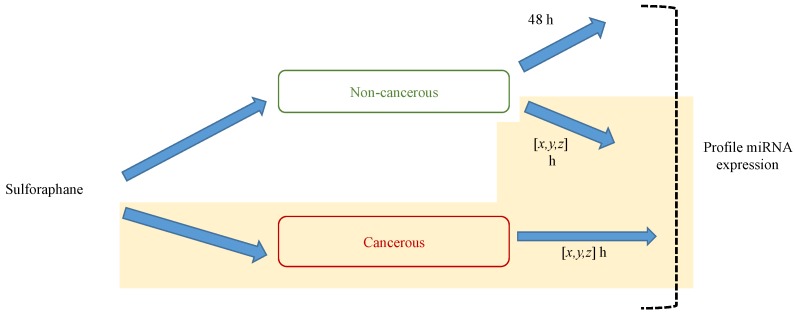
A basic overview of the miRNA-profiling experiments carried out by Slaby et al. and suggested further experiments. Slaby et al. treated non-cancerous colonic cell lines with sulforaphane, then profiled differences in miRNA expression at 48 h. Possible further experiments involving colorectal cancer cell lines and additional time points are illustrated and highlighted in yellow.

Therefore, further experiments to profile the modulation of miRNA expression at different time points, and in a cancerous colorectal cell line, could add significantly to the findings of Slaby et al. as illustrated in Figure 6. It would also be prudent to further examine miRNAs that are reportedly differentially expressed according to the wide-scale profiling process, using single-target assays, in order to rule out the possibility of them being artefacts of the former. Encouragingly, a human study reportedly showed that the consumption of 160g broccoli/day by participants was able to alter blood miRNA expression profiles, thus indicating that systemic regulation of miRNA expression in vivo is possible by doses of SFN obtainable from typical consumption of broccoli, although it cannot be ruled out that the observed modulations were mediated by components of broccoli other than SFN, such as fibre, and selenium and/or other micronutrients [78].

### 5.1. Mechanisms of Sulforaphane-Mediated Modulation

The possible mechanisms by which SFN may modulate miRNA expression are wide-ranging and could involve downstream effects of the SFN-mediated modulation of histone deacetylase (HDAC) activity, redox status, inflammatory signalling, miRNA-processing protein expression, and induction of Nrf2. For example, SFN is known to inhibit the activity of several HDACs [34] including HDAC3, which itself transcriptionally repressed the pro-apoptotic miR-15a-5p/16-1-5p cluster in mantle cell lymphoma cells [79]. SFN (15 µM) was also reported to directly inhibit HDAC3 activity in colorectal cancer HCT-116 cells, thereby inhibiting their proliferation, whilst having no similar effect in non-cancerous colonic CCD-841 cells [41]. SFN is also known to affect redox status—via its acute pro-oxidant and/or Nrf2-inducing effects—which can affect miRNA expression in various ways. For example, oxidative stress has been reported to inhibit Dicer activity in JAR trophoblast cells [80], to inhibit the Drosha partner protein DGCR8 [81], to activate Drosha via glycogen synthase kinase 3β activation [81], to inhibit adenosine diphosphate (ADP)-ribosylation and thus activity of AGO2 [82], and to induce ER stress such that induces an endoribonuclease (RNAse) called inositol-required enzyme 1α that can degrade the pre-miRNAs typically giving rise to miR-17-5p, miR-34a-5p, miR-96-5p and miR-125b-5p [81]. Interestingly, SFN-mediated Nrf2 induction may have additional, redox-independent consequences, since the 5’ flanking regions of certain miRNA genetic loci possess the antioxidant responsive element (ARE), as is found in the promoter regions for antioxidant protein-coding genes [83]. Nrf2 was reported to transcriptionally downregulate miR-29b-3p via ARE binding [83], but to upregulate the transcription of the mir-125b-1 and mir-29b-1 pre-miRNAs in acute myeloid leukaemia cells [84].

**Figure 7:**
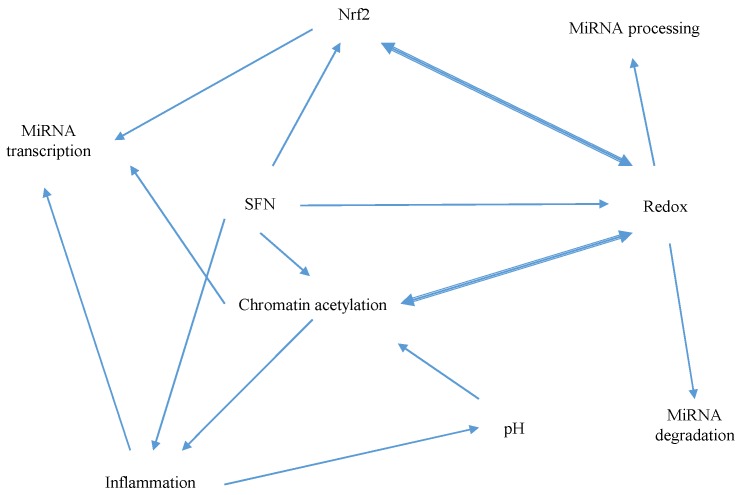
A diagram to illustrate the complexity of the network of cross-interactions between potential mechanisms of SFN-mediated miRNA modulation.

The anti-inflammatory effects of SFN are likely to also play a role in SFN-mediated miRNA modulation; an inflammatory medium containing TNF, IL-6, IL-8, and IL-1β was reported to upregulate miR-155-5p in several breast cancer cell lines, and miR-146a-5p in the HCT-15 and HCT-116 colorectal cancer cell lines [85]. Finally, proteins involved in miRNA biogenesis, including RNA polymerase II, Dicer, Drosha, DGCR8, Exportin 5, Ldbr and AGOs, are all potentially susceptible to SFN-mediated modulation. Further complicating the picture are considerations that HDAC activity, redox status, inflammatory signalling, miRNA-processing protein expression and Nrf2 can all cross-interact, as illustrated in Figure 7. For example, ROS can inhibit HDACs and activate histone acetyltransferases [86], whilst reciprocally, the ROS-generating DUOX nicotinamide adenine dinucleotide phosphate (NADPH) oxidases tend to be hypermethylated in lung cancer cell lines [87]. Inflammatory processes tend to reduce extracellular pH [88], which can promote H3 and H4 deacetylation [89]. Conversely, the HDAC inhibitor ITF2357 was reported to inhibit inflammatory cytokine expression in LPS-stimulated peripheral blood mononuclear cells [90].

### 5.2. Interaction of MicroRNAs with Pathogenesis

The potential for miRNAs to interact with colorectal cancer pathogenesis at different stages is vast.

#### 5.2.1. Interaction with Classic Vogelstein-Model Pathogenesis

**Figure 8:**
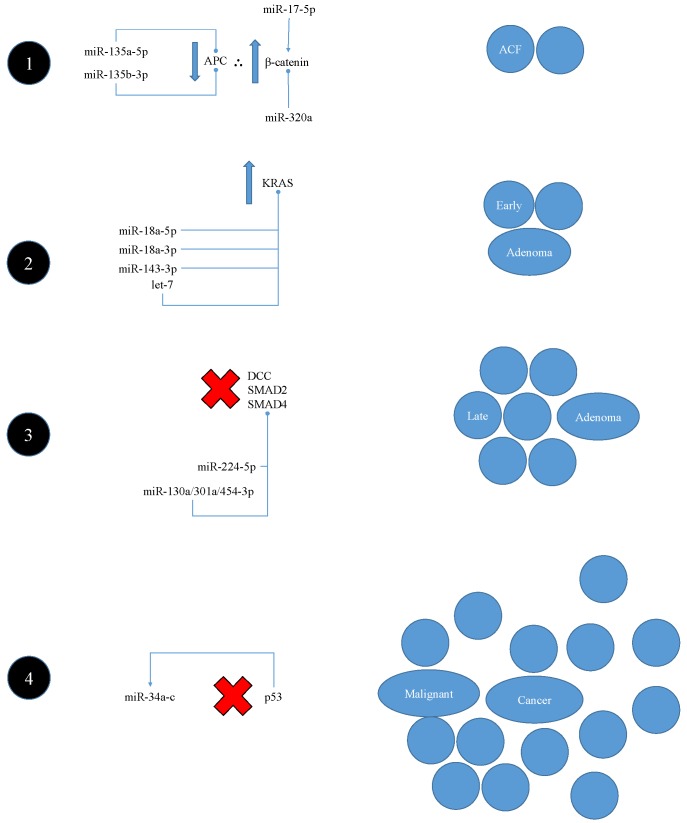
An illustration of the classic Vogelstein model of colorectal carcinogenesis and the reported potential interactions of miRNAs with each stage. (1) A reduction in APC activity derepresses β-catenin, which promotes cell proliferation and stem cell renewal. (2) Increased KRAS activity further promotes proliferation and the formation of an early adenoma. (3) The tumour suppressor genes DCC, SMAD2, and SMAD4 are lost, resulting in progression to a late adenoma. (4) The loss of p53 facilitates the eventual progression of the late adenoma to a malignant cancer.

According to the canonical Vogelstein model of colorectal cancer pathogenesis [91]—which is summarised in Figure 8—tumorigenesis typically begins with a reduction in adenomatous polyposis coli (APC) activity, resulting in increased β-catenin activity. β-catenin facilitates the formation of an aberrant crypt focus (ACF) via the upregulation of cell proliferation and stem cell renewal. There are two miRNAs commonly overexpressed in colorectal cancer specimens—miR-135a-5p and miR-135b-3p—which are both able to repress the translation of the APC protein [92], whilst miR-17-5p has been reported to also promote β-catenin activity [93]. Conversely, miR-320a has been shown to repress β-catenin and thereby inhibit tumour growth [93]. The next stage of pathogenesis according to the Vogelstein model is the upregulation of KRAS activity—believed to typically result from hyperactivating KRAS mutations. This leads to further acceleration of cell proliferation and the progression of the ACF to an early adenoma [91]. MiR-18a-5p, miR-18a-3p, miR-143-3p, and several let-7 miRNAs have all been demonstrated to translationally repress KRAS [92,93]. The following stage of pathogenesis according to the Vogelstein model is the loss of the apoptotic *DCC* gene and the TGF-β-driven tumour suppressor genes SMAD2 and SMAD4, and subsequent progression from an early to a late adenoma [91]. 

Interestingly, miR-224-5p and the miR-130a/301a/454-3p family have all been reported to directly repress SMAD4, whilst miR-106a-5p and miR-21-5p have been shown to repress the transforming growth factor (TGF)-β receptor II [93]. The final stage of pathogenesis according to the Vogelstein model, in which the tumour develops into a malignant cancer, is the loss of p53 activity—typically via the mutational inactivation of *TP53* [91]. Tumour suppression by p53 is partly mediated by its ability to induce the miR-34a-c family, including miR-34a-5p, which can repress the HDAC sirtuin 1 [92]. This miRNA is typically induced in response to DNA damage in a largely p53-dependent manner, and—along with other members of its family—tends to be deleted or hypermethylated in colorectal cancer specimens [93]. 

#### 5.2.2. Alternative Pathogenesis Model Interaction

**Figure 9:**
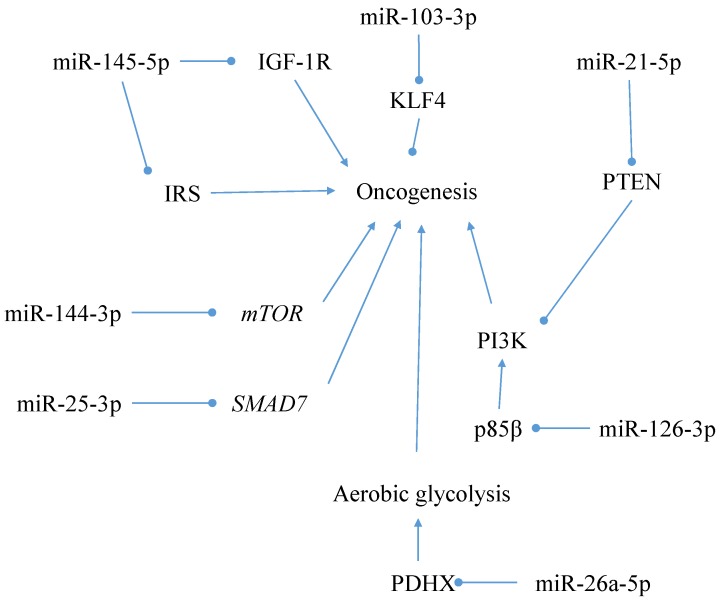
A diagram illustrating the interaction of miRNAs with genes and pathways regulating the pathogenesis of colorectal cancer.

As illustrated in Figure 9, there are many other miRNAs with the potential to interact with colorectal carcinogenesis additional to those mentioned above. For example, miR-144-3p and miR-25-3p are reported to repress the mTOR and SMAD7 oncogenes, respectively, the latter of which may inhibit TGF-β’s tumour suppressive functions [93]. MiR-145-5p is able to inhibit growth factor-induced proliferation by repressing the insulin-like growth factor-1 receptor and insulin receptor substrate, whilst miR-126-3p can repress p85β—a promoter of the oncogenic phosphoinositide 3-kinase (PI3K) signalling pathway [92]. MiR-21-5p can conversely upregulate this oncogenic pathway by repressing phosphate and tensin homolog (PTEN), and miR-103-3p can repress the tumour suppressor gene KLF4 [92]. MiR-26a-5p has been shown to be able to interact with cancer cell metabolism in the form of promoting aerobic glycolysis (i.e., the Warburg effect) by repressing pyruvate dehydrogenase protein X component, and thus the mitochondrial synthesis of acetyl-coenzyme A from pyruvate [94]. The Warburg effect can metabolically enhance the accumulation of intermediates that are involved in macromolecular synthesis and are thus required in abundance for rapid proliferation [95].

To summarise, there is much published evidence to suggest that miRNAs are involved in the pathogenesis of colorectal cancer, and that SFN is likely to modulate miRNA expression in the colorectum. Based upon existing knowledge regarding SFN’s bioactivity, there are many plausible mechanisms by which SFN might modulate miRNA expression. There are also many reports of specific miRNAs regulating tumour suppressor genes and/or oncogenes known to be involved in colorectal carcinogenesis. Together, these premises present a strong case for the further investigation of SFN-mediated miRNA modulation in cancerous and/or non-cancerous cell lines, and its possible implication for SFN’s potential to interact with colorectal cancers at different stages and in various contexts.

## 6. MicroRNA Assay Methods

### 6.1. Array-Based Methods

There are several types of wide-scale miRNA expression profiling methods that are frequently used, including the miRNA RT-qPCR array technique employed by Slaby et al. for which the TaqMan Low Density Array kit and human Megaplex RT Primer Pool v3.0 were used [44]. Such methods run hundreds of RT-qPCRs in parallel—each specific for a different miRNA—and by nature are relatively fast and economical with regards to required RNA sample input and overall cost. An alternative array-type approach involves the use of a miRNA array hybridisation chip—a medium coated with probes of sequence antisense to those of known miRNAs, to which miRNAs in samples can be hybridised, and which generate signals in response to the quantity of material bound to each specific probe [96]. Similarly to the RT-qPCR array method, this also tends to be relatively fast and economical. However, both of these approaches come with certain caveats. The range of detectable miRNAs is limited to those known at the time of development of the particular assay kit(s) used, which is an issue given that the database of known human miRNAs continues to grow; miRBase v.14 was released at the beginning of 2010 and catalogued 894 human miRNAs, whilst by the release of miRBase v.20 in the middle of 2013 there were 2555, and the current release, v.21, catalogues 2588 [97]. These approaches also come with sensitivity limitations.

### 6.2. Cloning and Deep Sequencing

An approach different to those described above, and with a number of advantages, is to clone all of the miRNAs present in a given sample into libraries, and then subject the libraries to deep sequencing, the data from which are analysed to evaluate the differential expression of known miRNAs, as well as to identify potentially novel miRNAs [98]. Data can be retained and later re-mapped to updated lists of miRNAs from later releases of miRBase. The cloning-sequencing approach also tends to be more sensitive in terms of profiling the differential expression of known miRNAs. The typical workflow for such experiments tends to begin with the ligation of 5’- and 3’-adapter oligomers to all small RNAs present in samples, followed by the reverse transcription of all adapter-ligated RNA to complementary DNA (cDNA). The cDNA products are then amplified by PCR and then size-separated by PAGE, in order to separate cloned miRNAs from clones of different types of small RNA [99]. Cloned miRNAs are then subject to deep sequencing, and the data generated are normalised and analysed to identify known human miRNAs that are differentially expressed between different conditions.

One challenge presented by this process is the inevitable sequence-dependence bias towards certain miRNAs over others in the adapter-ligation step, which results in the “favoured” miRNAs being more abundantly cloned and thus appearing more abundant upon analysis of deep sequencing data [98]. Such ligation bias should not impact the apparent differential expression of any given miRNA across different samples, given that a bias towards a specific miRNA would occur equally across all samples if the same adapters are used. However, one issue of ligation bias is the potential for certain miRNAs to go undetected, if strongly “disfavoured” by the ligation process. In order to mitigate ligation bias, HD adapter pools were developed by the Dalmay laboratory by adding to then-existing Illumina 5’- and 3’- adapters, four nucleotides at random, at the ligating ends [98]. This means that instead of single 5’- and 3’-adapters being used, pools consisting of 256 variants each are generated—each of which have different miRNA ligation bias profiles. These adapter pools have been demonstrated to dramatically increase miRNA coverage and to enable the detection of novel miRNAs [98]. A method of miRNA library construction using Dalmay HD adapters has been published by Xu et al. [99].

The main disadvantages of miRNA cloning-sequencing methods vs. the previously discussed array-based approaches are the greater cost and time involved, both for the construction and the sequencing of the libraries. However—where viable with regards to time and economics—investment in the cloning-sequencing approach could be worthwhile for the potential additional knowledge regarding differential miRNA expression obtained. It is also conceivable that overall costs may continue to decrease over time as a result of ongoing technological development, as per recently observable trends [100].

### 6.3. Comparison of Approaches to MicroRNA Profiling

A brief comparison of the above-discussed types of approach to the wide-scale profiling of miRNA expression is provided in Table 2.

## 7. Summary

Colorectal cancer poses an increasingly important health burden globally, with apparent links to diet that are unsurprising, given the liability of colorectum to be exposed to ingested compounds and products of the gut microbiota. Cruciferous vegetables such as broccoli and cauliflower are inversely correlated with colorectal cancer risk more strongly than other vegetables, and this is believed to be at least partially attributable to the isothiocyanates obtained by consuming these vegetables, such as SFN from broccoli. Isothiocyanates have been widely studied and shown to have anti-inflammatory, antioxidant, and cytoprotective effects through well-studied mechanisms, such as the induction of Nrf2. However, numerous anti-cancer effects have been demonstrated in vitro and in vivo that cannot be solely attributed to Nrf2 induction, particularly those acting to suppress advanced cancer cell proliferation. Since it is apparent that the effects of SFN are wide-ranging and complex—especially so in cancer—it is clear that further knowledge regarding its bioactivities would aid in the development of chemopreventive and/or chemotherapeutic strategies based upon it. 

MiRNAs are known to translationally regulate the expression of least 30% of protein-coding genes in humans and to play major roles in health and development, particularly in carcinogenesis. Their biogenesis and activities are highly complex both in their nature and their potential for regulation. There exists ample evidence in the published literature for the involvement of miRNAs in colorectal carcinogenesis, and numerous reports exist of direct miRNA-mediated regulation of specific tumour suppressor genes and/or oncogenes. Based upon existing knowledge regarding SFN’s activity, there exist many plausible mechanisms by which SFN might modulate the expression of different miRNAs. Therefore, the case for further investigation of the roles of miRNAs in the anti-cancer effects of SFN is strong.

There are several types of approach to wide-scale miRNA expression profiling, including array-based methods involving RT-qPCR or complementary probe hybridisation. These are relatively fast and economical, but detectable miRNAs are limited to those known at a given point in time. MiRNA cloning-deep sequencing is an alternative approach that can confer greater sensitivity that is not limited to the detection of miRNAs known at a given point in time. The challenge of adapter-ligation bias toward certain miRNAs potentially encountered at the miRNA cloning stage has been addressed by the development of Dalmay HD adapters—a library construction protocol using these adapters has been published. The downsides of the library construction and sequencing approach are its greater cost, time, and/or labour requirements. However, it is likely to be more informative with regard to differential miRNA expression and thus worth considering where viable.

## Figures and Tables

**Table 1:** Listed examples of reportedly tumour suppressive, oncogenic and ambiguous miRNAs and their reported target genes.

MicroRNA	Reported Role in Colorectal Cancer	Reported Target Genes
let-7a-5p	Tumour Suppressive	NIRF [57]
miR-34a-5p		E2F3 [58]
miR-101-3p		PTGS2 [59]
miR-126-3p		PIK3R2 [60]
miR-143-5p		DNMT3A [61]; ERK5 [62]
miR-195-5p		BCL2 [63]
miR-200a-3p		CTNNB1 [64]
miR-150-5p		MUC4 [65]; MYB [66]
miR-451a		MIF [67]
miR-17-5p	Oncogenic	P130 [68]
miR-92a-3p		BIM [69]
miR-23a-3p		MTSS1 [70]
miR-27a-3p		ZBTB10 [71]
miR-135b-5p		TGFβR2, DAPK1, APC [72]
miR-9-5p	Ambiguous	CDH [73]; TM4SF1 [74]
miR-21-5p		Pdcd4 [75]; CDC25A [76]; TGFβR2 [77]

**Table 2:** A brief comparison of array-based and library construction-sequencing-based approaches to wide-scale miRNA expression profiling.

Type of Method	Cost	Relative Time Required	MiRNA Detection
Array	Medium	Low	Limited to miRNAs known at the time of array development.
Library-Sequencing	High	High	Can identify and assay novel miRNAs, and retain data for re-mapping against updated miRNA databases.
